# Histone H3K4me1 strongly activates the DNase I hypersensitive sites in super-enhancers than those in typical enhancers

**DOI:** 10.1042/BSR20210691

**Published:** 2021-07-06

**Authors:** Yujin Kang, Jin Kang, AeRi Kim

**Affiliations:** Department of Molecular Biology, College of Natural Sciences, Pusan National University, Busan 46241, Korea

**Keywords:** DNase I hypersensitive site, eRNA, H3K27ac, H3K4me1, histone depletion, Super-enhancer

## Abstract

Super-enhancers (SEs), which consist of multiple enhancer elements, are occupied by master transcription factors and co-activators, such as Mediator, and are highly acetylated at histone H3K27. Here, we have characterized the SEs in terms of DNase I hypersensitive sites (DHSs) by analyzing publicly available chromatin immunoprecipitation (ChIP)-seq and DNase-seq data of K562 cells and compared with the DHSs in typical enhancers (TEs). DHSs in the SEs were highly marked by histone H3K4me1 than DHSs in TEs. Loss of H3K4me1 by the deletion of catalytic domains in histone methyltransferases MLL3 and MLL4 remarkably decreased histone H3K27ac and histone H3 depletion at SE DHSs than at TE DHSs. The levels of enhancer RNA (eRNA) transcripts and mRNA transcripts from the putative target genes were notably reduced at and near SE DHSs than TE DHSs following H3K4me1 loss. These results indicate that histone H3K4me1 is a marker for DHSs in SEs and that this modification has a more significant impact on the activation of SE DHSs than TE DHSs.

## Introduction

Enhancers are *cis*-regulatory elements that regulate cell type-specific gene expression [1,2]. They are often classified into super-enhancers (SEs) and typical enhancers (TEs) depending on their enrichment of Mediator or histone H3K27ac and the clustering of other enhancer elements within 12.5 kb [3–5]. Compared with TEs, SEs tend to cover longer regions, 10–60 kb, and are found to be fewer in number, by one to two orders of magnitude in various cell lines and tissues [3,5,6]. SEs are highly occupied by master transcription factors, such as Klf4, Esrrb, Nr5a2 and Prdm14 in embryonic stem cells and Myogenin and MyoD in myotubes, conferring biological roles in controlling cell identities [3,5,7]. In addition, SEs have been reported to promote oncogenic transcription that enhances cell survival and proliferation in tumorigenesis [4,8,9].

Enhancers have specific histone modifications, H3K4me1 and H3K27ac [10,11]. While histone H3K27ac is remarkable in active enhancers, histone H3K4me1 is present in both primed (paused) enhancers and active enhancers. Loss of H3K4me1 by the mutation or depletion of histone methyltransferases MLL3 (KMT2C) and MLL4 (KMT2D) negatively affects H3K27ac at enhancers and results in transcriptional inhibition of target genes in mammalian cells [12–14]. H3K4me1-containing mononucleosomes are efficiently bound and remodeled by BAF (SWI/SNF) chromatin remodeling complex *in vitro* [15]. Enhancer RNAs (eRNAs) are transcribed in an H3K4me1-dependent manner [12,16]. These findings provide insight into the fundamental roles of H3K4me1 in activating enhancers.

SEs have been characterized at the cluster level, including multiple enhancer elements. Here, to characterize SEs in terms of enhancer elements, we have analyzed publicly available DNase-seq and chromatin immunoprecipitation (ChIP)-seq data for histone H3K27ac and MED1, a subunit of the Mediator complex, in K562 cells. Chromatin structure at DNase I hypersensitive sites (DHSs) present in the SEs was analyzed and compared with the structure of DHSs in TEs. Based on the comparison results, the roles of histone H3K4me1 were explored in histone H3K27ac, histone H3 depletion and eRNA transcription at both SE DHSs and TE DHSs using ΔMLL3/4 K562 cells where the catalytic domains of MLL3 and MLL4 were deleted. Our study shows that H3K4me1, which is remarkable at DHSs of SEs, has a more powerful role in activating SE DHSs rather than TE DHSs.

## Materials and methods

### Identification of SEs

SEs were identified using the Rank Ordering of Super-enhancers (ROSE) pipeline [5]. Briefly, putative enhancers were obtained by overlapping DNase-seq peaks and histone H3K27ac ChIP-seq peaks and by excluding CTCF binding peaks. Sites within ±1 kb of the transcription start site (TSS) were excluded from the putative enhancers. Individual enhancers within 12.5 kb were stitched together to form a large enhancer cluster. Enhancer clusters were then ranked using the H3K27ac ChIP-seq signals that were subtracted by input signals. On the other hand, enhancer clusters were ranked by MED1 ChIP-seq signals. The x-axis point at which tangent to a scaled graph has one of the slope used as a cutoff; stitched enhancers above the slope were classified as SEs.

### ChIP-seq analysis

The ChIP-seq analysis was carried out as described [17]. ChIP-seq raw reads were qualified by excluding input reads with poor quality values (quality score 20) and then aligned to the human reference (hg19) genome using Bowtie2 [18]. Aligned reads were filtered using a minimum MAPQ quality score 20 and sorted by chromosomal coordinates, and then potential PCR duplicates were removed. Peak regions were identified by MACS2 using input data as controls [19]. The thresholds of the enrichment q-value were set as 0.05 for the narrow peak and 0.1 for the broad peak. Bigwig files were generated using bamCoverage or bamCompare tools and then visualized to heatmaps and average profiles. All public NGS data were obtained from the Gene Expression Omnibus (GEO) database. Raw reads were aligned to the hg19 genome for consistent data analysis. GEO accession numbers for these datasets were as follows: GSM2054697 for H3K4me1, GSM2054696 for H3K27ac, GSM2439223 for CTCF, GSM2574768 for MED1, GSM1003608 for GATA-1 and GSM623516 for DNase-seq. Control input for each ChIP-seq dataset was analyzed in a similar way.

### Deletion of the catalytic domains in MLL3 and MLL4 using CRISPR/Cas9

Single-guide RNA (sgRNA) sequences targeting the SET domains in MLL3 and MLL4 were designed using online tools (http://crispr.mit.edu). Two complementary oligos containing sgRNA sequences (EGFP oligo F; 5′-CACCGGGGCGAGGAGCTGTTCACCG-3′, EGFP oligo R; 5′-AAACCGGTGAACAGCTCCTCGCCCC-3′, MLL3_SET oligo F; 5′-ACCGTTCGAAACGAAGTAGCCAAC-3′, MLL3_SET oligo R; 5′-AAACGTTGGCTACTTCGTTTCGAA-3′, MLL4_SET oligo F; 5′-CACCGGGCCACCTCGTTCCGAATGA-3′, MLL4_SET oligo R; 5′-AAACTCATTCGGAACGAGGTGGCCC-3′) were phosphorylated and annealed as previously described [20]. Annealed oligos for the MLL3 SET domain were inserted into pLH-spsgRNA2 vector (Addgene #64114) [21] and annealed oligos for the MLL4 SET domain were inserted into lentiCRISPRv2 vector (Addgene #52961) [22]. The vectors were cloned in Stbl3 bacteria, prepared using the Plasmid Midi Kit (Qiagen), and transfected into 293FT cells using the Virapower packaging mix (Invitrogen) and Lipofectamine 2000 (Invitrogen). Lentiviruses with the MLL4 sgRNA were harvested and transduced into K562 cells in the presence of 6 µg/ml polybrene. The transduced cells were selected using 2 µg/ml puromycin and seeded in 96-well plates to produce clones. Lentiviruses with the MLL3 sgRNA were transduced into K562 cells where the MLL4 SET domains were deleted. The transduced cells were selected using 500 µg/ml hygromycin. Deleted regions were sequenced by TA cloning and/or by mapping of NGS data (BAM files) (Supplementary Figure S1A). Genomic sequences flanking the MLL3/4 SET domains were amplified by PCR (Supplementary Figure S1B).

### ChIP

The ChIP assay was performed as previously described [23]. Cells (1 × 10^7^) were cross-linked in 1% formaldehyde and digested using MNase. Fragmented chromatin was reacted with the antibodies and recovered using protein A agarose beads. DNA was purified by phenol extraction and ethanol precipitation. The antibodies used in the ChIP experiment were H3 (ab1791), H3K4me1 (ab8895) and H3K27ac (ab4729) from Abcam.

### ChIP DNA library preparation and sequencing

Ten nanograms of ChIPed DNA or input DNA was processed using the NEBNext Ultra II DNA Library Prep Kit (New England Biolabs #E7103S) according to the manufacturer’s instructions. Briefly, DNA was repaired at the ends, ligated with adaptors, selected in the 200 bp size using NEBNext sample purification beads, and amplified with the adaptor primers. After purification, the final libraries were quantified using a Qubit dsDNA HS assay (Invitrogen) and sequenced on an Illumina NovaSeq 6000 system at 100 base paired-end reads as previously described [24].

### RNA library preparation and sequencing

Total RNA was extracted using the RNeasy Plus Mini Kit (Qiagen) according to the manufacturer’s instructions and qualified using a Qubit Fluorometer (RNA IQ > 7). After depleting ribosomal RNA (rRNA) using the NEBNext rRNA Depletion Kit (New England Biolabs #E6350L), RNA was reverse transcribed using the NEBNext Ultra II Directional RNA Library Prep Kit (New England Biolabs #E7765S) as suggested by the manufacturer. This procedure included fragmentation, priming using random primers, first-strand cDNA synthesis, second-strand cDNA synthesis, cDNA end repair, adaptor ligation, amplification using adaptor primers and purification using beads. Final libraries were quantified using a Qubit dsDNA HS assay (Invitrogen) and sequenced on an Illumina NovaSeq 6000 system at 100 base paired-end reads.

### RNA-seq analysis

Quality scores of the sequenced reads were assessed using a quality control tool. Input-read ends with poor quality values were removed from raw reads using Trimmomatic [25]. The remaining reads were aligned to the hg19 genome using STAR [26]. The exon annotation GTF file was obtained from the UCSC database. Each of the aligned reads was counted for each gene ID (Entrez) using featureCounts [27]. CPM values above 1 were considered meaningful transcription levels in genes.

### Statistical analysis

*P*-values were calculated using the two-tailed Student’s *t* test (*P<0.05). Boxplot data were evaluated using a two-tailed Student’s *t* test between the two groups of enhancers. All boxplots indicate log_2_-transformed (CPM + 1).

## Results

### SEs consist of more DHSs than TEs

**Figure 1:**
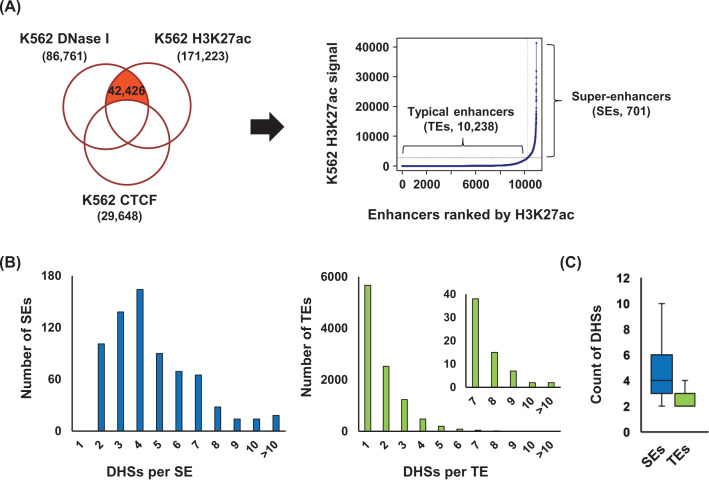
Identification of SEs in K562 cells (**A**) Putative enhancer elements were identified by overlapping DNase-seq peaks (*n*=86761) and H3K27ac ChIP-seq peaks (*n*=171223) and excluding CTCF-binding sites (*n*=29648) (left). The enhancer elements (*n*=42426) were clustered using the ROSE algorithm, and 701 SEs and 10238 TEs were identified depending on H3K27ac signals (right). (**B**) DHSs within both the SEs (left, blue) and TEs (right, light green) were counted. (**C**) The boxplots display the number of DHSs in each of the two sets of enhancers, except where the TEs presented with only a single DHS.

*Cis*-regulatory elements, such as enhancers, promoters and insulators, are identified by hypersensitivity to DNase I attack in a chromatin context. Here we identified our enhancer elements by overlapping the DNase-seq peaks and histone H3K27ac ChIP-seq peaks in erythroid K562 cells and then by excluding CTCF binding sites using NGS data obtained from the Encyclopedia of DNA Elements (ENCODE) curated by UCSC (Figure 1A, left). This identified 42426 putative enhancer elements, which were then evaluated using the ROSE algorithm that uses H3K27ac signals (Figure 1A, right) or MED1 signals (Supplementary Figure S2A) to rank these elements. Enhancer clusters above 1 of the slope value in the tangent to curve equation were classified as SEs (*n*=701), and clusters below this point were considered TEs (*n*=10238). The ratio of SEs to TEs was 6.85%, similar to the ratio in other cell types [6]. When we counted the DHSs per enhancer cluster, SEs showed to have 4.72 of DHSs, while more than 55% of the TEs included just a single DHS and those with multiple DHSs (44.6%) included an average of 2.76 sites per cluster (Figure 1B,C). MED1 signal based classification produced a similar pattern (Supplementary Figure S2B,C). These results indicate that SEs have higher number of DHSs than TEs.

### Histone H3K4me1 is enriched at SE DHSs

**Figure 2:**
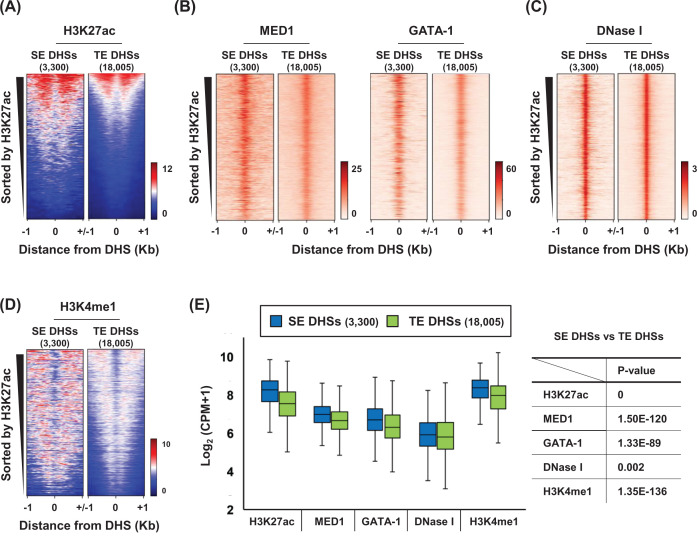
Chromatin structure at the DHSs in SEs and TEs of K562 cells (**A**) Heatmaps show histone H3K27ac signals at the DHSs in the SEs (*n*=3300) and TEs (*n*=18005). MED1 and GATA-1 signals (**B**), DNase I sensitivity (**C**) and histone H3K4me1 signals (**D**) at the DHSs were visualized using heatmaps. Color scales indicate the relative signal intensity. DHSs were sorted by H3K27ac signals. (**E**) Boxplots depict the average signal for H3K27ac, MED1, GATA-1, DNase I and H3K4me1 within ±1 kb of DHSs in the SEs and TEs. P-values were calculated using a two-tailed *t* test. The y-axis indicates log_2_-transformed (CPM + 1).

To characterize DHSs present in SEs, we compared the chromatin structure at 3300 DHSs present in SEs with the structure at 18005 DHSs present in TEs using public ChIP-seq and DNase-seq data in K562 cells. SE DHSs were highly marked by histone H3K27ac than TE DHSs as shown by heatmaps (Figure 2A). Occupancy of MED1 and GATA-1, an erythroid master transcription factor, was greater in SE DHSs (Figure 2B), although DNase I sensitivity was slightly higher in SE DHSs than in TE DHSs (Figure 2C). Notably, histone H3K4me1 was more enriched in SE DHSs than TE DHSs (Figure 2D). These chromatin features were visualized in the genome browser for SEs and TEs (Supplementary Figure S3). The ChIP-seq data from Figure 2A–D were graphed as boxplots with statistical analysis (Figure 2E). These results indicate that histone H3K4me1, in addition to MED1 and GATA-1, is greater in SE DHSs than TE DHSs. Similar results were obtained for DHSs present in SEs and TEs classified by MED1 signals (Supplementary Figure S4).

### Loss of H3K4me1 affects H3K27ac and H3 depletion at SE DHSs than TE DHSs

**Figure 3:**
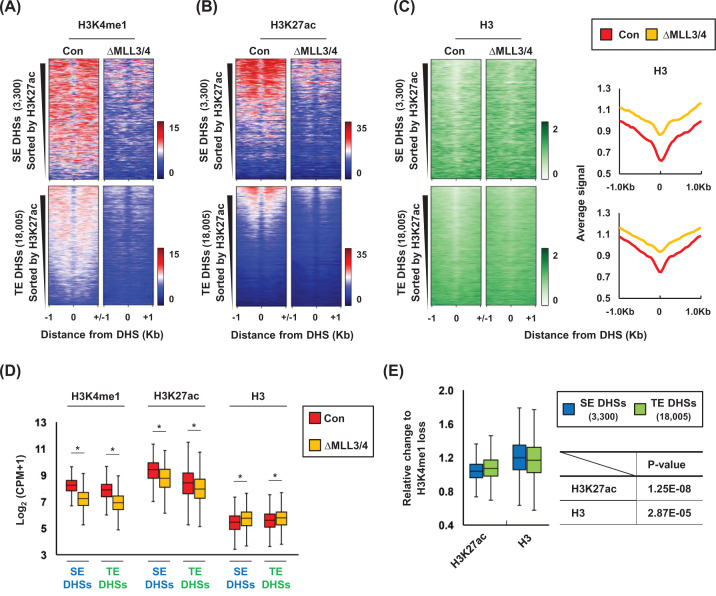
Chromatin structure at SE DHSs and TE DHSs in control and ΔMLL3/4 K562 cells Signals for H3K4me1 (**A**) and H3K27ac (**B**) were drawn using heatmaps at the DHSs in the SEs and TEs in control (Con) and ΔMLL3/4 K562 cells (ΔMLL3/4). (**C**) Histone H3 occupancy at the DHSs was visualized using heatmaps and average profiles. Color scales indicate the relative signal intensity on heatmaps. DHSs were sorted by H3K27ac signals. (**D**) Signals for H3K4me1, H3K27ac and H3 within ± 1 kb of the DHSs were described in boxplots (*P*-values; H3K4me1 SE DHSs = 0, H3K4me1 TE DHSs = 0, H3K27ac SE DHSs = 1.40E-137, H3K27ac TE DHSs = 5.50E-252 and H3 SE DHSs = 2.68E-39, H3 TE DHSs = 3.15E-76). (**E**) Boxplots display the ratio of H3K27ac decrease relative to H3K4me1 loss and the ratio of H3 increase to H3K4me1 loss within ±1 kb of the DHSs in SEs and TEs. *P*-values were calculated using a two-tailed *t* test. Relative change in H3K27ac = log_2_ {(ΔMLL3/4 H3K27ac CPM + 1)/(Con H3K27ac CPM + 1)} − log_2_ {(ΔMLL3/4 H3K4me1 CPM + 1)/(Con H3K4me1 CPM + 1)}, relative change in H3 = log_2_ {(ΔMLL3/4 H3 CPM + 1)/(Con H3 CPM + 1)} − log_2_ {(ΔMLL3/4 H3K4me1 CPM + 1)/(Con H3K4me1 CPM + 1)}. *P<0.05. P-values were calculated using the two-tailed t test' in panel (E) of Figure 3.

To elucidate the role of histone H3K4me1 in activating the chromatin structure at SE and TE DHSs, we deleted the DNA sequences encoding catalytic domains of MLL3 and MLL4 using the CRISPR/Cas9 technique in K562 cells (ΔMLL3/4). Deletion of 12 or 14 bp was sequenced in exon 56 of the Mll3 alleles and deletion of 13 or 21 bp was analyzed in exon 51 of the Mll4 alleles (Supplementary Figure S1). ΔMLL3/4 reduced H3K4me1 at DHSs of both SEs and TEs, as shown by ChIP-seq analysis (Figure 3A). Reduction of H3K4me1 resulted in a decrease in histone H3K27ac and an increase in histone H3 occupancy at these DHSs (Figure 3B,C). These changes were presented as boxplot graphs with statistical processing (Figure 3D). In addition, when we compared the impact of H3K4me1 loss using the ChIP-seq data, the decrease in H3K27ac and the increase in H3 occupancy were greater in SE DHSs than TE DHSs (Figure 3E). The great effects of H3K4me1 loss were observed in SE DHSs classified by MED1 signals (Supplementary Figure S5). Thus, these results indicate that histone H3K4me1, required for H3K27ac and histone H3 depletion at DHSs of enhancers, has a more powerful role in activating SE DHSs compared with TE DHSs.

### eRNA transcription from SE DHSs is more sensitive to the loss of H3K4me1 than eRNA transcription from TE DHSs

**Figure 4:**
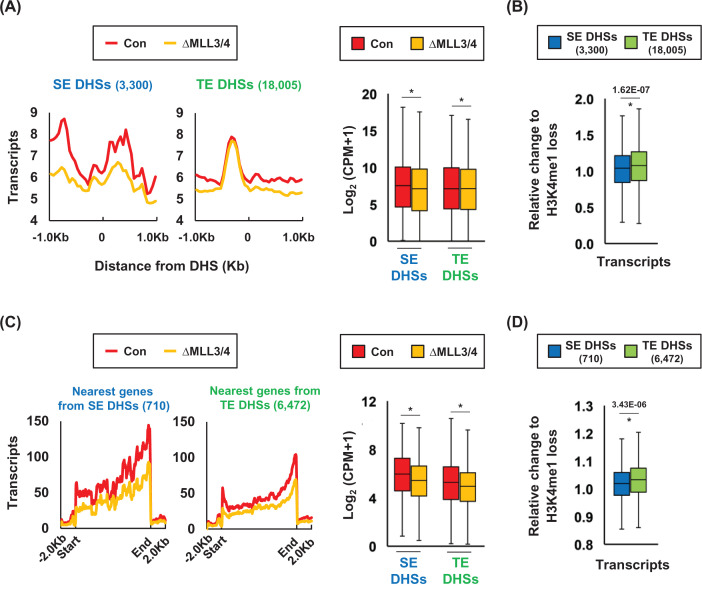
eRNA and putative target gene transcription at and near SE DHSs and TE DHSs in control and ΔMLL3/4 K562 cells (**A**) eRNA transcription within ±1 kb of the DHSs in SEs and TEs was visualized using average profiles and boxplots for control and ΔMLL3/4 K562 cells (*P*-values; SE DHSs = 9.61E-08, TE DHSs = 2.36E-13). (**B**) The change of eRNA level relative to H3K4me1 loss was shown in the boxplots. (**C**) Transcription of the gene nearest to the DHSs of SEs and TEs was evaluated in control and ΔMLL3/4 cells (*P*-values; SE DHSs = 1.13E-05, TE DHSs = 3.18E-13). Length of the nearest genes was scaled to 10 kb. (**D**) Boxplots depict the ratio of gene transcription to H3K4me1 loss at the DHSs. *P*-values were calculated using a two-tailed *t* test. Relative change in eRNA = log_2_ {(ΔMLL3/4 eRNA CPM + 1)/(Con eRNA CPM + 1)} − log_2_ {(ΔMLL3/4 H3K4me1 CPM + 1)/(Con H3K4me1 CPM + 1)}, relative change in gene transcription = log_2_ {(ΔMLL3/4 transcripts CPM + 1)/(Con transcripts CPM + 1)} − log_2_ {(ΔMLL3/4 H3K4me1 CPM + 1)/(Con H3K4me1 CPM + 1)}. *P<0.05. P-values were calculated using the two-tailed t test' in panel (D) of Figure 4.

We examined the role of histone H3K4me1 in eRNA transcription from SE DHSs by analyzing total RNA-seq data in control and ΔMLL3/4 cells. The level of eRNAs was higher in SE DHSs than TE DHSs (Figure 4A). The levels were reduced following the loss of H3K4me1. The reduction was more severe in SE DHSs than in TE DHSs (Figure 4B). Similar results were observed in the transcription of the genes nearest to the DHSs; the gene nearest to SE DHSs were highly transcribed than the gene nearest to TE DHSs, and the gene transcription associated with SE DHSs was more sensitive to the loss of H3K4me1 than the other gene transcription (Figure 4C,D). The severe effect of H3K4me1 loss on eRNA and mRNA transcription was observed in SE DHSs classified by MED1 (Supplementary Figure S6). Thus, this analysis implies that H3K4me1 strongly affects eRNA and mRNA transcription at and near SE DHSs compared with the transcription at and near TE DHSs.

## Discussion

**Figure 5:**
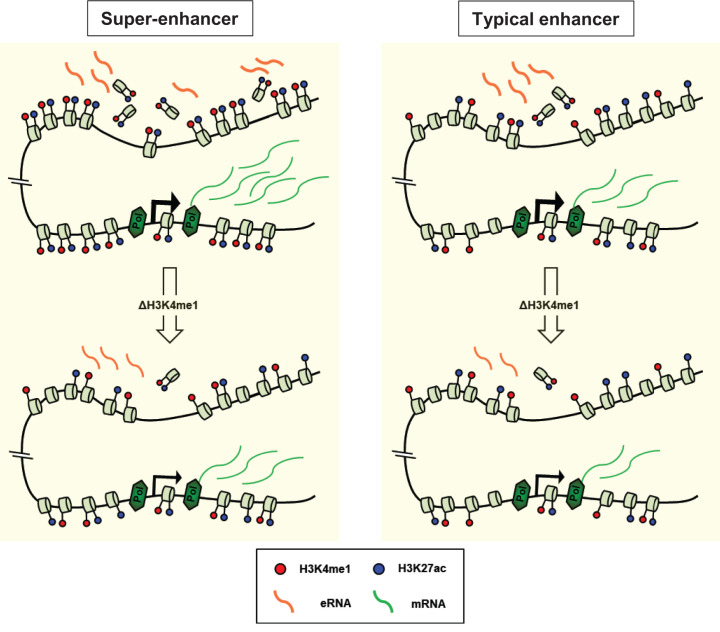
Effects of histone H3K4me1 loss on the DHSs of SEs and TEs Results from each figure were combined and visualized in diagrams. DHSs present in SEs were shown to have more active chromatin structure than DHSs present in TEs. The loss of H3K4me1 has a greater impact on H3K27ac, histone H3 depletion, eRNA transcription and mRNA transcription at or near the DHSs in SEs than TEs.

Our comparative analysis shows that SEs consist of a higher number of enhancer elements than TEs. These enhancer elements that were identified as DHSs have a more active chromatin structure in SEs than TEs. Notably, histone H3K4me1 was more remarkable in SE DHSs than TE DHSs in K562 cells. The loss of this modification significantly affected histone H3K27ac, histone H3 depletion and eRNA transcription at both SE and TE DHSs, but its effects were more pronounced for the SE DHSs. Thus these findings suggest that histone H3K4me1 plays a more remarkable role in the activation of SE DHSs than TE DHSs (Figure 5).

SEs contain many DHSs that have active histone modifications and are associated with MED1 and GATA-1 in K562 cells. It is unclear whether the number of DHSs is critical for enhancer activity or their function. However, active chromatin features of SE DHSs are likely to contribute to them. Hypersensitivity to DNase I and strong histone depletion at SE DHSs indicate an accessible chromatin structure favorable for transcription factor and co-activator binding. This might associate with hyperacetylation at histone lysine residues that weaken the interaction between histones and DNA [28,29]. Histone H3K27ac at enhancers has been known to be required for eRNA transcription [30,31]. High levels of eRNA transcription from the perspective of SEs, not a view of DHSs, have been reported in macrophage cells [32].

Histone H3K4me1 is a well-known histone marker for enhancers. Our analysis shows that this modification is more remarkable for SE DHSs than for TE DHSs in K562 cells. H3K4me1 appears to contribute to the activation of SE DHSs than TE DHSs. This could be due to the absolutely higher level of H3K4me1 in SE DHSs. H3K4me1 has been known to be recognized by the PHD domain [15]. The PHD domain is present in histone acetyltransferases such as CBP and p300 [33,34] and the ACF1 and DPF3 subunits of ATP-dependent chromatin remodeling complexes [35,36]. Thus, H3K4me1 is likely to play a role in generating histone H3K27ac and nucleosome depletion by recruiting the co-activators. H3K27ac is recognized by BRD4, which recruits RNA polymerase II into enhancers [37,38] and is required for eRNA transcription [30]. The transcription of eRNAs might be linked to the transcription of target genes, which is compatible with our results that both eRNA and mRNA transcription are affected by H3K4me1 loss. Further studies on the relationship between H3K4me1 and the recruitment of ATP-dependent chromatin remodeling complexes might explain the powerful role of H3K4me1 in activating SE DHSs.

## Supplementary Material

Supplementary Figures S1-S6

## Data Availability

The ChIP-seq and RNA-seq datasets are available under the GEO accession number: GSE147826. Data were analyzed according to the ENCODE guideline.

## Abbreviations

ChIP: chromatin immunoprecipitation
CTCF: CCCTC-binding factor
DHS: DNase I hypersensitive site
ENCODE: Encyclopedia of DNA Elements
eRNA: enhancer RNA
GEO: Gene Expression Omnibus
ROSE: Rank Ordering of Super-enhancer
SE: super-enhancer
sgRNA: single-guide RNA
SWI/SNF: ATP-dependent chromatin remodeling complexes
TE: typical enhancer
