# Supplementary material for: Histone H3K4me1 strongly activates the DNase I hypersensitive sites in super-enhancers than those in typical enhancers
Source: Biosci Rep. 2021 Jul 6;41(7):BSR20210691. doi: 10.1042/BSR20210691 (PMC8264496; doi:10.1042/BSR20210691)
Supplement: Supplementary Figures S1-S6 [file BSR-2021-0691_supp.pdf]

Figure S1. Deletion of catalytic domains in the MLL3 and MLL4 genes using CRISPR/Cas9 system.

A

MLL3 exon 56

WT : TTGAGTACATCGGGACTATCA**TTCGAAACGAAGTAGCCAACAGG**AAAGAGAAGCTTTATGAGTC

Allele 1 : TTGAGTACATCGGGACTATCA**TTCGAAAC**------------**AGG**AAAGAGAAGCTTTATGAGTC

Allele 2 : TTGAGTACATCGGGACTATCA**TTCGAAACGAA**--------------AGAGAAGCTTTATGAGTC

MLL4 exon 51

WT : CAATGGTTATCGAGTACATTGGCA**CCATCATTCGGAACGAGGTGGCC**AACCGGCGGGAGAAAAT

Allele 1 : CAATGGTTATCGAGTACATTGGCA**C**-------------**GAGGTGGCC**AACCGGCGGGAGAAAAT

Allele 2 : CAATGGTTATCGAGTAC---------------------**GAGGTGGCC**AACCGGCGGGAGAAAAT

B

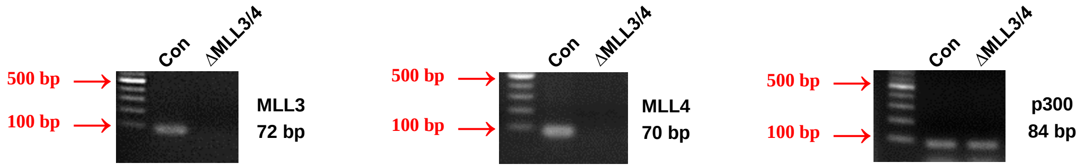

Figure S1. Deletion of catalytic domains in the MLL3 and MLL4 genes using CRISPR/Cas9 system. (A) SET domains of the MLL3 (exon 56) and MLL4 (exon 51) were targeted by single guide RNAs (sgRNAs). sgRNA sequences and PAM sites were indicated by black bold and red bold bases, respectively. Deleted sequences were presented by dash line in two alleles of the MLL3 and MLL4 genes. **(B)** Genomic DNA was amplified to detect deletions with the primers designed for gRNA sequences and control primers. PCR products were visualized on agarose gels. The sequences of primers used are as follows. MLL3 F: 5′ CGAGACATTGAGAAACACACCA3′, MLL3 R: 5′ CCTGTTGGCTACTTCGTTTCG 3′, MLL4 F: 5′ CTCTATGCAGCCAAGGACCT 3′, MLL4 R: 5' CGTTCCGAATGATGGTGCCA 3', p300 F: 5' CTTCTGACAAAACCGTGGAAG 3', p300 R: 5' TTTGGATCCACGAGGAGAAG 3'.

Figure S2. Identification of super-enhancers in K562 cells.

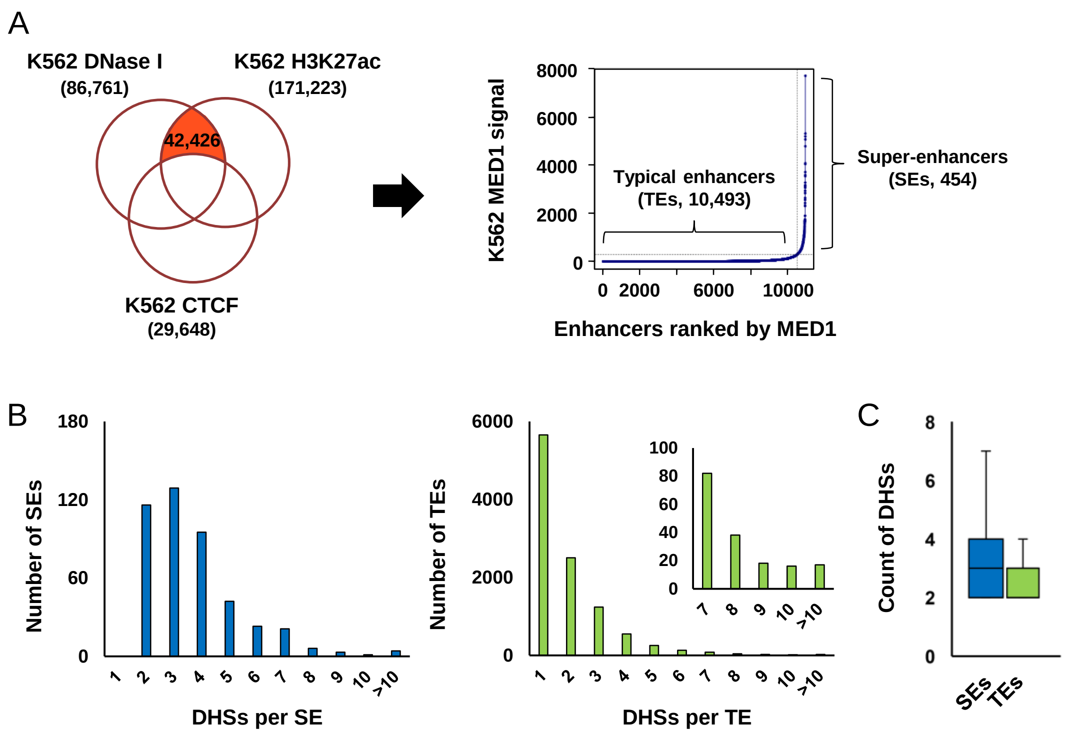

Figure S2. Identification of super-enhancers in K562 cells. (A) Putative enhancer elements were identified by overlapping DNase-seq peaks (n = 86,761) and H3K27ac ChIP-seq peaks (n = 171,223) and excluding CTCF binding sites (n = 29,648) (left). The enhancer elements (n = 42,426) were clustered using the ROSE algorithm, and 454 super-enhancers (SEs) and 10,493 typical enhancers (TEs) were identified depending on MED1 signals (right). **(B)** DNase I hypersensitive sites (DHSs) within both the SEs (left, blue) and TEs (right, light green) were counted. **(C)** The boxplots display the number of DHSs in each of the two sets of enhancers, except where the TEs presented with only a single DHS. 3.80 of DHSs at average were found in SEs, whereas TEs consist of single DHS at 53.9% and multiple DHSs at 46.1% with 2.96 at average.

Figure S3. The genome browser of SEs and TEs.

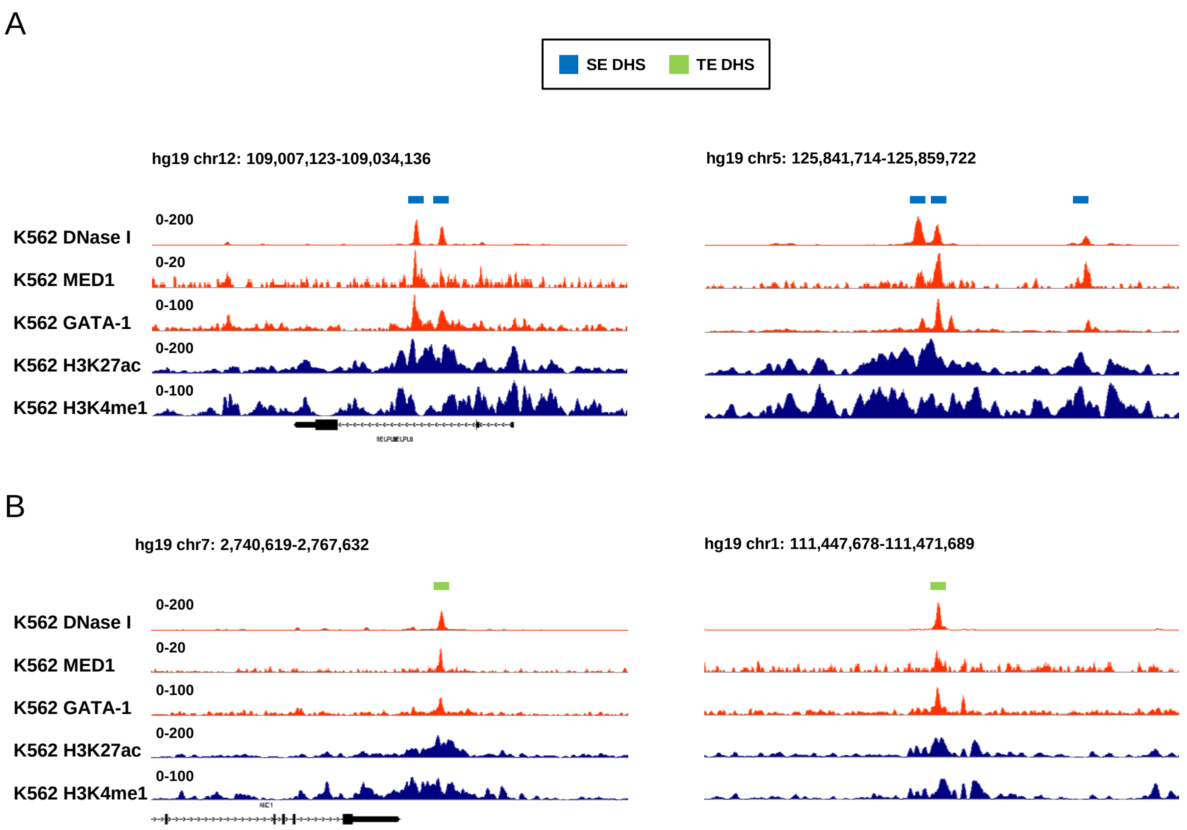

Figure S3. The genome browser of SEs and TEs. The IGB genome browser tracks represent enrichment profiles of DNase I, MED1, GATA-1, H3K27ac and H3K4me1 at the top 10% of SEs **(A)** and at the top 10% of TEs **(B)**. DHSs of SEs and TEs are represented with blue bars and light green bars, respectively.

Figure S4. Chromatin structure at DHSs present in SEs and TEs that are classified by MED1 signals in K562 cells.

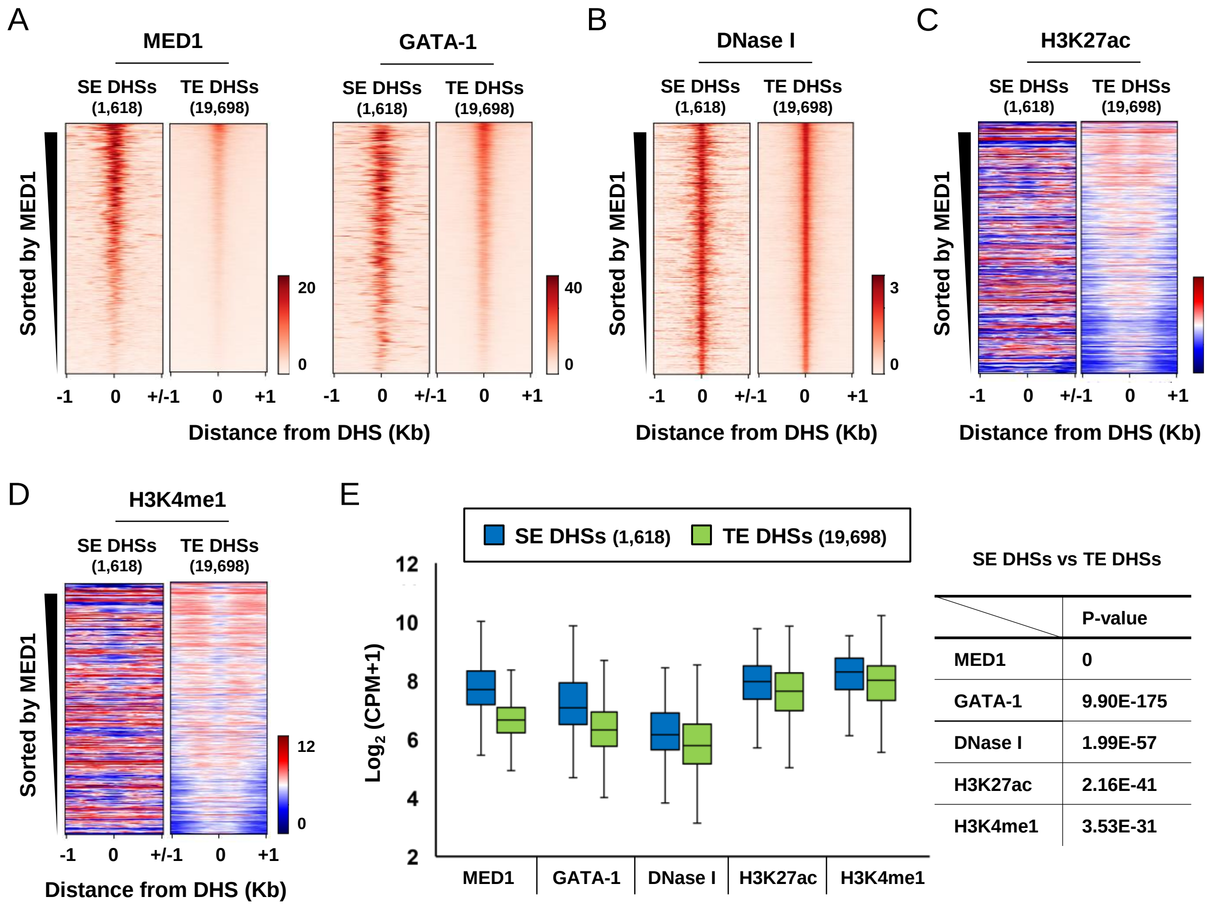

Figure S4. Chromatin structure at DHSs present in SEs and TEs that are classified by MED1 signals in K562 cells. (A) Heatmaps show signals of MED1 and GATA-1 at DHSs present in SEs and TEs that are classified by MED1 signals. Signals of DNase I sensitivity **(B)**, histone H3K27ac **(C)** and H3K4me1 **(D)** at the DHSs of SEs and TEs were presented by heatmaps. Color scales indicate the relative signal intensity on heatmaps. DHSs were sorted by MED1 signals. **(E)** Boxplots depict average signal of MED1, GATA-1, DNase I, H3K27ac and H3K4me1 at ± 1 Kb of DHSs in SEs and TEs. P-values were calculated using a two-tailed t test. The y axis indicates $\log_2$-transformed (CPM + 1).

Figure S5. Chromatin structure at DHSs within SEs and TEs that are classified by MED1 signals in control and △MLL3/4 cells.

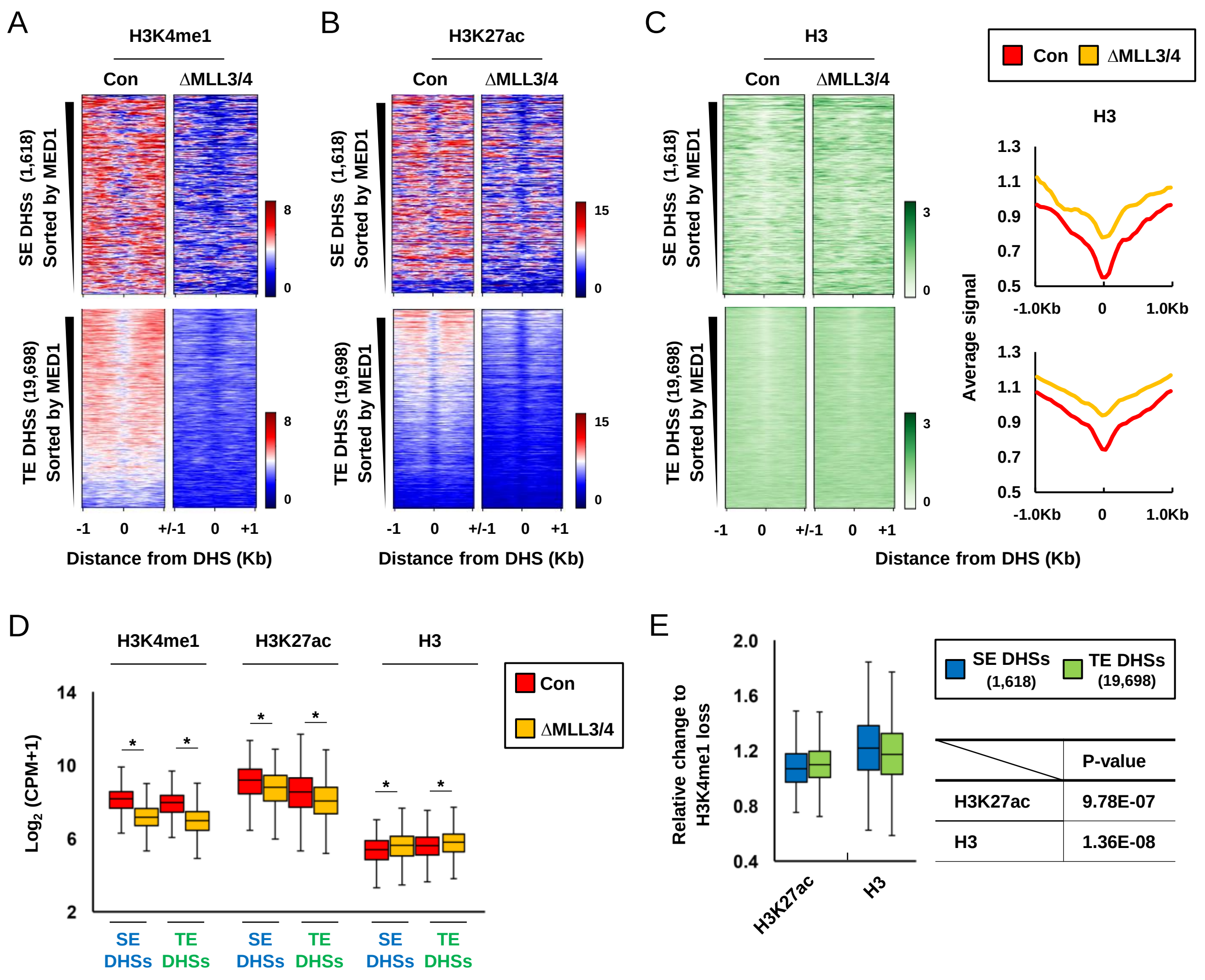

Figure S5. Chromatin structure at DHSs within SEs and TEs that are classified by MED1 signals in control and △MLL3/4 cells. Signals of H3K4me1 **(A)** and H3K27ac **(B)** were drawn by heatmaps at DHSs present in SEs and TEs that are classified by MED1 signals in control (Con) and △MLL3/4 K562 cells (△MLL3/4). **(C)** Histone H3 occupancy at the DHSs was shown by heatmaps and average profiles. Color scales indicate the relative signal intensity on heatmaps. DHSs were sorted by MED1 signals. **(D)** Signals of H3K4me1, H3K27ac and H3 at ± 1 Kb of DHSs were presented in boxplots (P-values; H3K4me1 SE DHSs = 7.50E-204, H3K4me1 TE DHSs = 0, H3K27ac SE DHSs = 9.01E-21, H3K27ac TE DHSs = 0 and H3 SE DHSs = 2.35E-13, H3 TE DHSs = 6.44E-98). **(E)** Boxplots display the ratio of H3K27ac decrease to H3K4me1 loss and ratio of H3 increase to H3K4me1 loss at ± 1 Kb of DHSs in SEs and TEs. P-values were calculated using a two-tailed t test. Relative change of H3K27ac = $\log_2$ {(△MLL3/4 H3K27ac CPM + 1) / (Con H3K27ac CPM + 1)} - $\log_2$ {(△MLL3/4 H3K4me1 CPM + 1) / (Con H3K4me1 CPM + 1)}, relative change of H3 = $\log_2$ {(△MLL3/4 H3 CPM + 1) / (Con H3 CPM + 1)} - $\log_2$ {(△MLL3/4 H3K4me1 CPM + 1) / (Con H3K4me1 CPM + 1)}.

Figure S6. Transcription of eRNA and putative target genes at and near SE DHSs and TE DHSs that are classified by MED1 signals in control and △MLL3/4 cells.

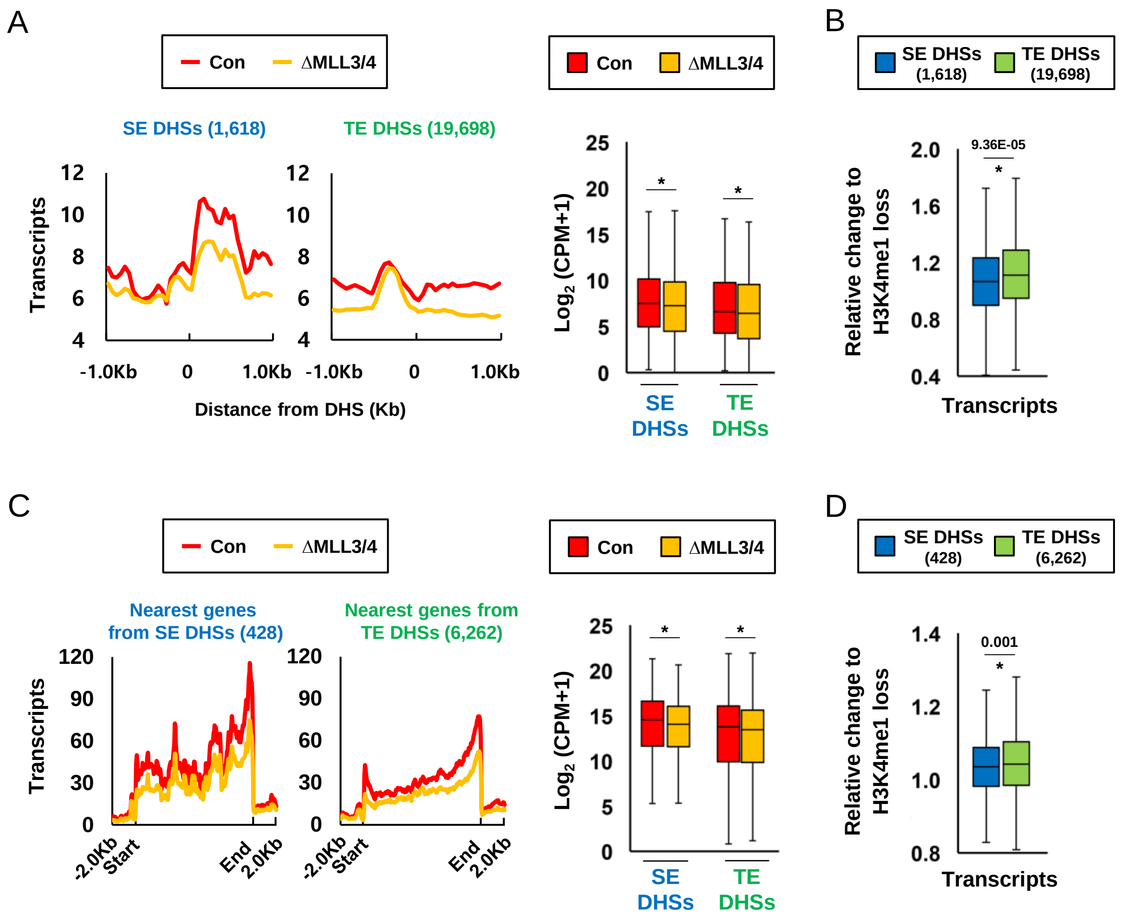

Figure S6. Transcription of eRNA and putative target genes at and near SE DHSs and TE DHSs that are classified by MED1 signals in control and △MLL3/4 cells. (A) eRNA transcription at ± 1 Kb of DHSs in SEs and TEs that are classified by MED1 signals was depicted by average profiles and boxplots in control and △MLL3/4 K562 cells (P-values; SE DHSs = 4.11E-05, TE DHSs = 2.17E-15). **(B)** Ratio of eRNA decrease to H3K4me1 loss was presented by boxplots at the DHSs of SEs and TEs. **(C)** Transcription of the nearest genes from DHSs of SEs and TEs was measured in control and △MLL3/4 cells (P-values; SE DHSs = 0.004, TE DHSs = 6.46E-04). Length of the nearest genes was scaled to 10 Kb. **(D)** Boxplots show ratio of gene transcription decrease to H3K4me1 loss at DHSs. P-values were calculated using a two-tailed t test. Relative change of eRNA = $\log_2$ {(△MLL3/4 eRNA CPM + 1) / (Con eRNA CPM + 1)} - $\log_2$ {(△MLL3/4 H3K4me1 CPM + 1) / (Con H3K4me1 CPM + 1)}, relative change of gene transcription = $\log_2$ {(△MLL3/4 transcripts CPM + 1) / (Con transcripts CPM + 1)} - $\log_2$ {(△MLL3/4 H3K4me1 CPM + 1) / (Con H3K4me1 CPM + 1)}.
